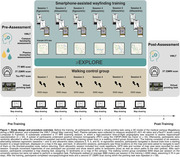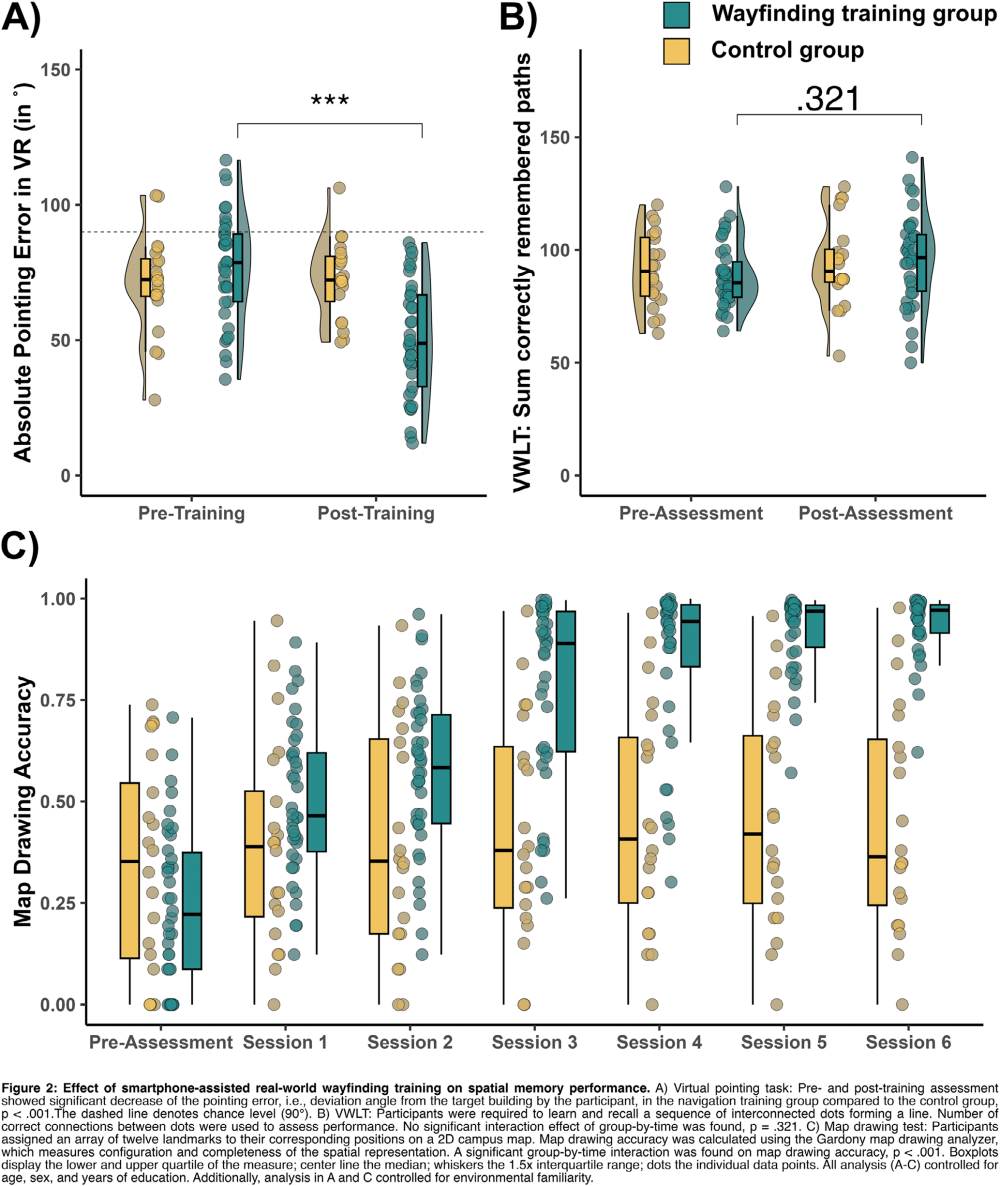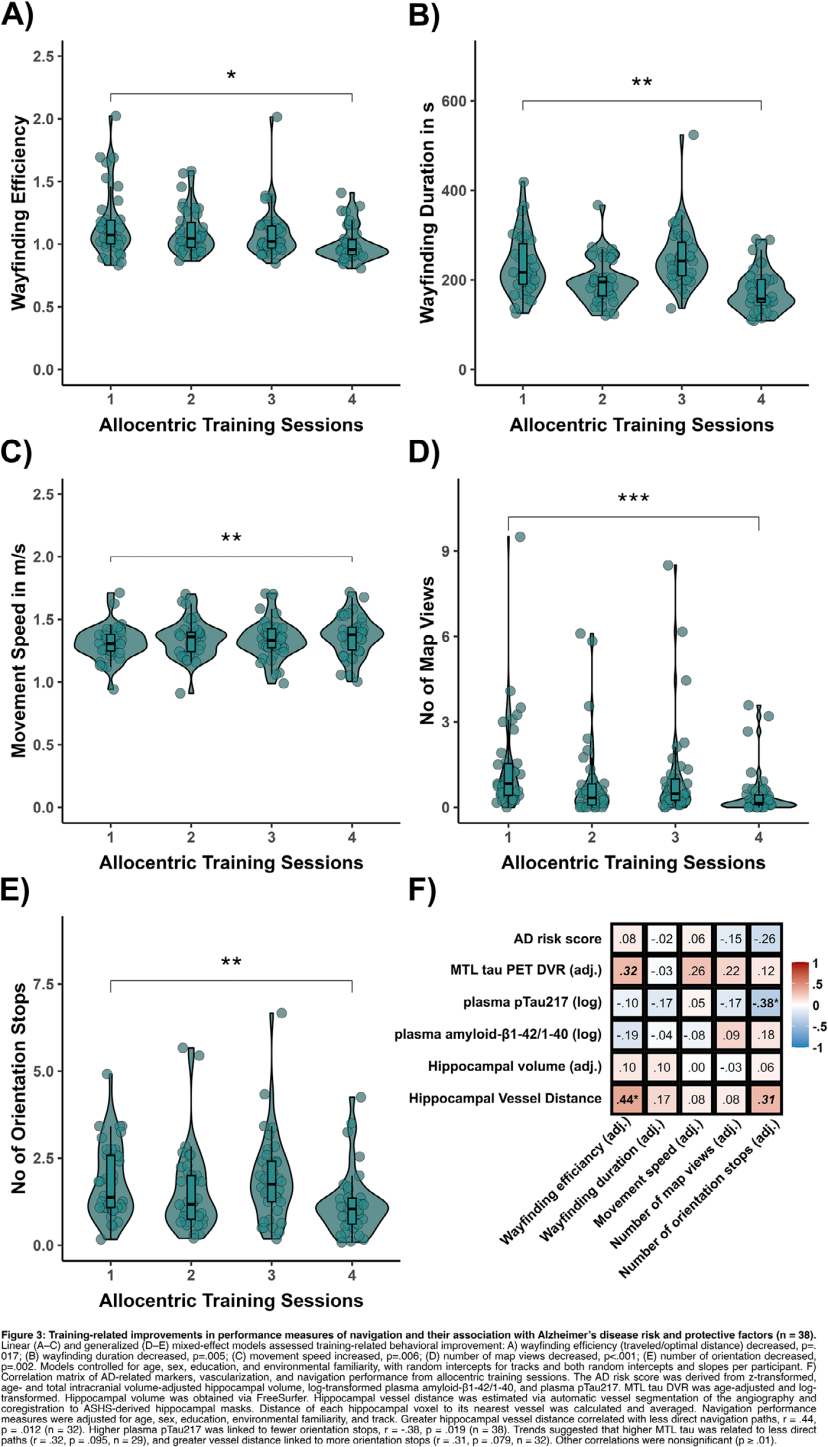# Hippocampal vascularization is associated with greater efficiency during a remote real world wayfinding training in older adults

**DOI:** 10.1002/alz70860_105004

**Published:** 2025-12-23

**Authors:** Jonas Marquardt, Niklas Vockert, Niklas Behrenbruch, Beate Schumann‐Werner, Anne Hochkeppler, Anna‐Therese Büchel, Eóin N. Molloy, Svenja Schwarck, Larissa Fischer, Enise I. Incesoy, Berta Garcia‐Garcia, Hendrik Mattern, Barbara Morgado, Hermann Esselmann, Andrew W. Stephens, Andreas Schildan, Henryk Barthel, Osama Sabri, Jens Wiltfang, Michael C. Kreissl, Emrah Düzel, Esther Kuehn, Stefanie Schreiber, Anne Maass, Nadine Diersch

**Affiliations:** ^1^ German Center for Neurodegenerative Diseases (DZNE), Magdeburg, Germany; ^2^ Institute of Cognitive Neurology and Dementia Research (IKND), Otto‐von‐Guericke University, Magdeburg, Germany; ^3^ University Clinic for Psychosomatic Medicine and Psychotherapy, Otto‐von‐Guericke University Magdeburg, Magdeburg, Germany; ^4^ Division of Nuclear Medicine, Department of Radiology & Nuclear Medicine, Faculty of Medicine, Otto von Guericke University, Magdeburg, Germany; ^5^ Department of Biomedical Magnetic Resonance, Otto‐von‐Guericke University, Magdeburg, Germany; ^6^ Center for Behavioural Brain Sciences (CBBS), Magdeburg, Sachsen‐Anhalt, Germany; ^7^ Department of Psychiatry and Psychotherapy, University Medical Center Göttingen (UMG), Göttingen, Germany; ^8^ Piramal Imaging GmbH, Berlin, Germany; ^9^ Department of Nuclear Medicine, University of Leipzig, Leipzig, Germany; ^10^ Department of Psychiatry and Psychotherapy, University of Göttingen, Göttingen, Germany; ^11^ German Center for Neurodegenerative Diseases (DZNE), Goettingen, Germany; ^12^ Institute of Cognitive Neurology and Dementia Research (IKND), Otto‐von‐Guericke University, Magdeburg, Sachsen Anhalt, Germany; ^13^ Hertie Institute for Clinical Brain Research, Tübingen, Germany; ^14^ German Center for Neurodegenerative Diseases (DZNE), Tübingen, Germany; ^15^ Department of Neurology, Otto‐von‐Guericke University, Magdeburg, Germany; ^16^ Institute for Biology, Otto von Guericke University, Magdeburg, Germany

## Abstract

**Background:**

Alzheimer's Disease (AD) pathology accumulates early in the medial temporal lobe (MTL), crucial for spatial navigation. As spatial navigation is among the first cognitive functions affected by AD, it may benefit from targeted behavioral interventions. We investigated the potential of a novel smartphone‐assisted real‐world wayfinding training, tailored for healthy older adults, to improve their spatial abilities and explored associations with hippocampal vascularization and AD biomarkers.

**Method:**

38 cognitively healthy older adults (62–84 years; 18 females) participated in a 3‐week navigation training, using our smartphone application “Explore” (Figure 1). Training involved finding several locations displayed on a map in the medical campus area of Magdeburg, Germany, while GPS data were recorded. Pre‐ and post‐training, participants underwent fMRI, performed a pointing task in a virtual campus version, and completed the VWLT. At pre‐assessment, AD pathology was characterized by plasma sampling (Abeta1‐42/1‐40, Ptau217) and [18F]PI‐2620 PET in a subsample. Hippocampal vascularization was assessed by 7T angiography. Performance in the virtual pointing task and a map drawing test was compared to a control group (*n* = 20) who performed a walking task of equal length without a navigational component. Additionally, changes in different mobile wayfinding performance indicators and their associations with AD biomarkers and hippocampal vascularization (i.e., mean distance of hippocampus to surrounding vessels) were examined.

**Result:**

Performance in the pointing task and map drawing, but not in the VWLT (*p* = .321), significantly improved due to the training (all *p* < .001; Figure 2A C). The control group showed no improvements in navigation. Training benefits were also evident in the mobile data (all *p* ≤.017; Figure 3A‐E). Better wayfinding efficiency was associated with less vessel distance to hippocampus, r=.44, *p* = .012, and the number of orientation stops was negatively related to pTau217, r=‐.38, *p* = .019 (Figure 3F).

**Conclusion:**

We provide evidence that a remotely administered real‐world wayfinding training enhances wayfinding abilities and improves spatial memory in older adults. Importantly, hippocampal vascularization may benefit wayfinding efficiency. Higher pTau217 was related to fewer orientation stops during navigation. As a next step, potential mediating effects between vascularization and AD pathology on wayfinding performance will be investigated.